# The Importance of Intra-Organizational Networking for Younger Versus Older Workers: Examining a Multi-Group Mediation Model of Individual Task Performance Enhancement

**DOI:** 10.3389/fpsyg.2020.606383

**Published:** 2020-12-15

**Authors:** Beatrice I. J. M. Van der Heijden, Peter M. Kruyen, Guy Notelaers

**Affiliations:** ^1^Institute for Management Research, Radboud University, Nijmegen, Netherlands; ^2^School of Management, Open University of the Netherlands, Heerlen, Netherlands; ^3^Department of Marketing, Innovation and Organisation, Ghent University, Ghent, Belgium; ^4^Hubei Business School, Hubei University, Wuhan, China; ^5^Kingston Business School, Kingston University, London, United Kingdom; ^6^Department of Psychosocial Science, University of Bergen, Bergen, Norway

**Keywords:** age, intra-organizational networking, employability, individual task performance, multi-group analysis

## Abstract

The purpose of this paper is to investigate the effect of intra-organizational networking on individual task performance, via employability. Moreover, this study also examines whether this relationship differs for younger (<40 years) versus older employees (≥40 years). A self-report questionnaire was distributed among a sample of employees working in a range of different types of organizations (*n* = 374). We conclude that employability fully mediates the relationship between intra-organizational networking and individual task performance. However, this mediation effect did not vary between younger and older employees. This study extends past research by applying a human capital perspective (in particular, social capital) and life-span development frameworks for explaining employability and task performance enhancement across one’s working life. It provides useful insights for stimulating career development and individual performance growth, by means of social capital, herewith increasing the individual employee’s chance to survive in nowadays’ labor markets.

## Introduction

Contemporary careers are characterized by the continuous need to develop one’s occupational knowledge, abilities, and skills (i.e., competences) (Kuijpers et al., 2016; Forrier et al., 2018), and, through this, one’s job performance (Van der Heijden et al., 2009, 2020; De Vos et al., 2020). Concepts such as the boundaryless career (Arthur, 1994) and protean career (Hall, 2004; see also Briscoe and Hall, 2006; Sullivan and Arthur, 2006) reflected the idea that employees could not longer rely on one employer to develop a sustainable career (Iles, 1997), and have stressed the individual’s responsibilities for employability management throughout their career (Inkson and Arthur, 2001; Van der Heijden et al., 2020). That is, employees are expected to invest in their employability (or career potential) enhancement to safeguard the required level of their performance. In particular, nowadays, workers face an ample amount of challenges they have to deal with, such as ever-increasing market pressures, growing speed in developments (e.g., new technology and new production concepts), expanded globalization, leaner organizations (Hines et al., 2018), and, therefore, rapid changes in job requirements (Lazarova and Taylor, 2009; Baran and Woznyj, 2020). These developments urge workers to be highly adaptable (Sullivan et al., 1998; Van der Heijden et al., 2020), and necessitate that they continuously update their competences (Berntson et al., 2006; Nazar and Van der Heijden, 2012; Van der Heijde and Van der Heijden, 2014; Van der Heijden et al., 2016), after their formal education and initial occupational choice.

As a result, current scholarly career researchers are called to focus on possible antecedents that enable workers to protect and further enhance their employability (Van der Heijden et al., 2009; Cortellazzo et al., 2020), and through this their performance across the life-span (Ybema et al., 2017). This contribution focuses on the predictive validity of intra-organizational networking, being an important activity in nowadays’ career management, for workers’ employability, using a competence-based approach (Van der Heijde and Van der Heijden, 2006), and, through this, for their individual task performance. The advantage of this competence-based measurement approach or, also referred to, an input-based approach of employability (cf. more output-oriented ones; Vanhercke et al., 2014), comprising knowledge, skills and attitudes, or more general, competencies, is that is measures an individual worker’s career potential. In particular, on the one hand, when scholars use an output-based approach, they focus on indicators of employability, such as the individual’s perceptions of their chances to find new employment or for making a transition across different positions at the labor market (Vanhercke et al., 2014). On the other hand, when scientists use an input-based approach, they are enabled to disentangle the importance of different antecedents of employability, their interrelatedness, and to examine how workers can make progress in their employability enhancement (cf. Van der Heijde and Van der Heijden, 2006; Van der Heijden et al., 2018). Specifically, the competence-based operation by Van der Heijde and Van der Heijden (2006) refers to an individual’s capacities that enable his or her potential for permanent acquisition and fulfillment of employment, within or outside one’s current organization, for one’s present or new customer(s), and with regard to future prospects (p. 453).

In line with Bozionelos (2008), we have focused on intra-organizational networks in order to investigate the added value of an individual’s social capital (Seibert et al., 2001; Bozionelos, 2003; Dobrow and Higgins, 2005; Rodrigues et al., 2019), that is the structure and quality of all interpersonal ties within a particular working context (Adler and Kwon, 2002), in this case one’s working organization. As regards our outcome measure, we have chosen for task performance which can be defined as the competency (i.e., the proficiency) that an individual portrays with regard to their central job tasks (Campbell, 1990). We argue that a competence-based approach to employability, being the hypothesized mediator, is represented most optimal through a research model wherein employee performance is operationalized as task performance.

Notwithstanding the rich (empirical) literature about the value of social capital for individuals who possess it (see for instance Burt et al., 2000; Hirschi et al., 2018), to the best of our knowledge, this is the first empirical work that goes into the importance of networking in the light of employability enhancement, and, through this, on performance at work. In addition, we aim to investigate whether the strength of the relationships in this proposed mediation model are moderated by employee age. More specifically, we distinguish between “younger workers” and “over-forties” [(see Finkelstein and Farrell, 2007, p. 100) on the Age Discrimination in Employment Act (ADEA) (see also Van der Heijden et al., 2009)].

This article is structured as follows. First, in our theoretical framework, we present five research hypotheses which, taken together, accumulate in a moderated mediation model. Next, we provide the details of our research methodology. After reporting our main findings, we end this contribution with a discussion on the importance of intra-organizational networking to enhance both employability and performance, and we go into some limitations of this empirical work and recommendations for future research.

## Theoretical Framework

### Employability

The employability literature has expanded vastly over the past decennia, and contributions come from various disciplines such as labor economics, management science, and psychology (Thijssen et al., 2008; Forrier et al., 2015; Lo Presti et al., 2018). Next to the different perspectives that are brought along by the distinguished disciplines, the concept is studied on different levels, such as a societal, governmental, organizational, and individual level (Versloot et al., 1998). Employability is commonly defined as one’s ability to realize employment within and between employers over time (Forrier et al., 2009). Previous researchers have presented the phenomenon of employability as a personal resource (De Cuyper et al., 2012), a “personal asset” (Forrier et al., 2018) that employees should strive to acquire in order to effectively cope with the current labor market demands, and a set of competencies (Van der Heijde and Van der Heijden, 2006; Van der Heijden et al., 2018). In this contribution, the competence-based short-form operationalization of employability by Van der Heijde and Van der Heijden (2006) (see Van der Heijden et al., 2018) is used, in order to respond to the need to depart from the complex and constantly changing challenges the individual employee has to deal with in their working life. In the current labor market, employees need a broad package of knowledge and skills (Wright and Snell, 1998; Baran and Woznyj, 2020) throughout the life-span, that incorporates social and adaptive expertise (Rodriguez et al., 2002; Frie et al., 2019), on top of technical domain-specific knowledge. Therefore, aging, employability and the role of continuous development and learning in this regard, are high on the agenda of all parties involved in nowadays’ working organizations (Froehlich et al., 2014; Dello Russo et al., 2020). Van der Heijde and Van der Heijden (2006) considered employability to consist of five dimensions. First, they included *occupational expertise* as an important component for employability. Occupational expertise comprises the domain-related knowledge and skills in a certain area. Next, they argued that employees need to proactively map their surroundings and prepare themselves for possible changes in job and career requirements and conditions (*anticipation and optimization)* and, subsequently, adapt to them (*personal flexibility)* (Seibert et al., 1999; Fugate et al., 2004). Anticipation and optimization is defined as investing in preparing for and adapting to possible changes in one’s work, in a personal and creative manner, herewith striving for the best possible results. Personal flexibility refers to the capacity to adapt easily to all kinds of changes in the internal and external labor market that do not pertain to one’s immediate job domain. Furthermore, identification with the organization’s goals and the ability to work together with one’s peers, so-called *corporate sense*, is needed (Podsakoff et al., 1997) in order “to stay in the race.” Corporate sense is defined as one’s capability to perform well in different work groups, such as, organizations, teams, occupational communities and other networks. Sharing responsibilities, knowledge, experiences, feelings, credits, failures, goals, etc. Lastly, Van der Heijde and Van der Heijden (2006) named *balance* as a key competence – the ability to balance between one’s employer’s interests and one’s own interests, and to balance between reaching one’s own opposing work, career, and private interests.

In sum, the above-outlined five distinguished dimensions of competences are used in this contribution to define the amount of employability of individual workers. The domain-independent operationalization of employability by Van der Heijde and Van der Heijden (2006) comprises a valid and reliable multi-trait instrument with sound convergent and discriminant validity or distinctive power of the five scales. The five dimensions are not fully exclusive. In other words, they represent correlated aspects of employability (ibid.), measured through a unit-weighted set of items that are considered equally important.

After this outline on the competence-based operationalization of employability that has been used in this contribution, we will go into the added value of intra-organizational networking for fostering workers’ career potential.

### The Importance of Intra-Organizational Networking

Human capital theory (Becker, 1993) may be used to understand the investments of employers in their workers’ further career development. Pearce and Randel (2004) mentioned the “employability trend” in HRM wherein employers provide interesting jobs and opportunities to their staff in order to build up competences that can be used to develop a mobile career [see also Galunic and Anderson (2000) who referred to “generalized investments in employees”]. However, the responsibility to actively engage in developmental strategies to acquire new knowledge and skills (to build up new human capital or competences) and to actively participate in networks that can serve as conduits of industry, technological, and product knowledge (to build up their social capital) (McFadyen and Cannella, 2004; Spurk et al., 2019) rests principally on the shoulders of individual workers (Smith, 2010, p. 9–10). Therefore, the first objective of this study is to investigate whether networking within one’s organization is a useful strategy for employees to enhance their employability, and, as a result, their performance at work.

Social capital is located in a network of more or less durable social relations and may be defined as a resource that can facilitate certain actions (Pearce and Randel, 2004, p. 83). These social relations may provide the individual with access to new knowledge, valuable resources, and career opportunities (Nahapiet and Ghoshal, 1998; Spurk et al., 2019). A comprehensive conceptualization of social capital comprises network resources (see for example Seibert et al., 2001; Bozionelos, 2003; Baruch and Bozionelos, 2010). Network resources are those resources that individuals have at their disposal by means of their network ties, and may be characterized as either career-instrumental or socio-emotional (Bozionelos, 2015, p. 70; see for example Fombrun, 1982; Saint-Charles and Mongeau, 2009).

From the perspective of employability enhancement, we hypothesize that networking behaviors reflect the linkages employees build with others, and that may be valuable in the light of enhancing their career potential. In particular, intra-organizational networking behaviors may be defined as “individuals’ attempts to develop and maintain relationships with others in the organization who have the potential to assist them in their work or career” (see also Forret and Dougherty, 2004, p. 420; Ben-Hador and Eckhaus, 2018). We argue that employees who have strong social ties with others in their organization, and who belong to formal or informal networks that deal with issues that cross formal task groups (Hansen, 1999), are able to expand their knowledge and skills (Lin, 1999; Ben-Hador, 2016), increase their job performance, and, as such, to advance their career (see also Froehlich et al., 2015; Huang, 2017). In particular, networking with colleagues in both formal and informal professional bodies is a common way to expand one’s technological skills’ base (Benner, 2002; Miranda and Borges, 2019). Moreover, previous studies across different types of occupations already indicated that networking is associated with augmented skill sets, including tacit skills (Adams and Demaiter, 2008; Guo et al., 2018) and staying on top of industry news and innnovations (Smith, 2010; Paruchuri and Awate, 2017).

Based upon insights from human capital theory (Becker, 1993), we posit that investments made in human capital (i.e., knowledge and skills; forming the basis of workers’ competence-based employability) are associated with economic value, for instance in the form of task performance as portrayed by the individual employee, and that the latter is of direct use to achieve valued organizational outcomes (see also De Cuyper et al., 2014). Consequently, participation in networking as an individual career management strategy is of utmost importance as the main responsibility for protecting and fostering one’s employability and career success has shifted from the organization to the individual employee (Arthur and Rousseau, 1996; De Vos and Van der Heijden, 2015; De Vos et al., 2020; Van der Heijden et al., 2020). That is to say, developing interpersonal relationships through networking may be seen as a specific career strategy for building up expertise that is vital for managing one’s career (Arthur et al., 1995, 1999; Hirschi et al., 2018; Jacobs et al., 2019).

In the present contribution, we hypothesize that employability mediates the relationship between intra-organizational networking and individual task performance. In other words, we hypothesize that optimal performance at work requires that an individual employee possesses the occupational knowledge and skills (i.e., competences or career potential; employability) that are necessary to meet their organizational demands, and that these competences can be enhanced by investing in networking activities. Arocena et al. (2007) – based on their work in small- and medium-sized enterprises – already concluded that workers’ employability is positively associated with their capability of doing their tasks and with their motivation to make extra efforts, herewith resulting in a better task performance appraisal (see also Villanueva, 2005; Hahn and Kim, 2018). However, as scholarly research on the relationship between employability and individual task performance is scarce (Camps and Rodríguez, 2011; Hahn and Kim, 2018), the first contribution of this empirical work is to increase our knowledge on how to safeguard employees’ added value and their chances to survive in current labor markets.

Although full mediation is a possible outcome, earlier scholarly work in the domain of networking suggest the possibility of direct associations with task performance as well. Indeed, Wolff and Moser (2009), and Spurk et al. (2015), stated that networking behavior is a key factor in career development, including one’s performance (see also Thompson, 2005) and career-related outcomes, such as career optimism and career satisfaction (Volmer and Wolff, 2018). In a similar vein, Reiche (2012) found that social capital of inpatriates (i.e., subsidiary countries’ nationals who have moved to the headquarters of a multinational corporation) was related to the continuity of knowledge transfer from the headquarters toward the subsidiary after they had returned. Obviously, the latter may be seen as an important basis for increased job performance among staff members, herewith supporting our line of reasoning that employability (partially) mediates the relationship between intra-organizational networking and individual task performance.

**FIGURE 1:**
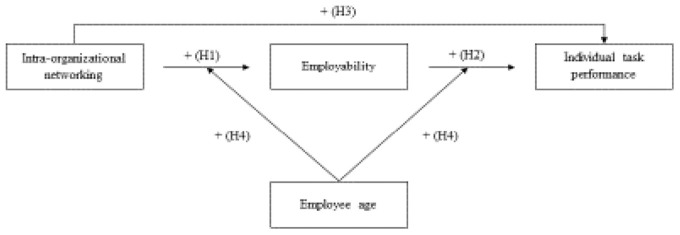
Hypothetical model.

Based on the outlines given above, we have formulated the following hypotheses (see Figure 1):

*Hypothesis 1:* Intra-organizational networking is positively related to employability.
*Hypothesis 2:* Intra-organizational networking is positively related to individual task performance.
*Hypothesis 3:* Employability is positively related to individual task performance.
*Hypothesis 4:* Employability (partially) mediates the relationship between intra-organizational networking and individual task performance.

### Examining a Multi-Group Mediation Model of Individual Task Performance Enhancement

Previously, career researchers already called for more empirical work studying differences in relationships between model variables for distinguished age groups (see for instance Van der Heijden et al., 2009, 2016), including scholarly work on contemporary employability approaches (Tisch, 2015). In this contribution, we build upon several life-span developmental theories and previous empirical work on aging and work-related outcomes. Life-span developmental perspectives are based upon the notion that patterns of change occur with aging, and that employee development involves adaptive processes, such as acquisition, maintenance, transformation, and attrition (Baltes et al., 1999). All in all, applying life-span developmental theories to the workplace elaborates on the assumption that adaptation over time involves self-regulation in order to cope with gains and losses that are characteristic for aging at work. Adopting such a perspective for a better understanding for the interplay between age and work is useful as most individuals spend a considerable part of their life-span at work, and have ample opportunities to portray these adaptive processes (see Truxillo et al., 2012a, b; Veth et al., 2019). Specifically, the second objective of our study is to investigate possible differences across age groups (in our case younger workers and over-forties) when we examine the relationship between intra-organizational networking and individual task performance, via employability.

Some scholars (see for instance Maurer, 2001; Farr and Ringseis, 2002; Armstrong-Stassen and Ursul, 2009) argued that Human Resource Management (HRM) policies and practices are especially important for protecting and further enhancing older workers’ career potential (Kanfer and Ackerman, 2004; Brooke and Taylor, 2005; Truxillo et al., 2014, 2015; Veth et al., 2019). In particular, as a result of the decline in fluid intelligence and the increase in crystallized intelligence with aging (Baltes et al., 1999), older employees are more likely to adopt specific strategies for maximizing gains and for minimizing losses by using available personal resources (Selective Optimization with Compensation: SOC theory; Baltes et al., 1999, see also Ebner et al., 2006; De Lange et al., 2011; Zacher and De Lange, 2011; Truxillo et al., 2012a, b; Vignoli et al., 2019). In other words, in order to offset age-related losses, people search for compensating strategies to protect their level of performance (see Truxillo et al., 2012b). Correspondingly, we argue that developmental opportunities at work, in our case, intra-organizational networking, are highly beneficial as they increase the employee’s ability to adopt and to fine-tune these strategies, and herewith their employability, especially for older employees (see also Van der Heijden et al., 2015; Veth et al., 2019).

Another important framework that might be beneficial in explaining possible moderating effects due to age is the Life-Span Theory of Control (Heckhausen et al., 2010). This theory states that with aging people are inclined to rely more on secondary control strategies. More specifically, “ … secondary control can foster development and enhance primary control by contributing to the selection of action alternatives throughout the life course; and when primary control is threatened or lost, secondary control strategies can help maintain or minimize losses in primary control as well as expand the potential for primary control without the individual having to physically engage the environment” (Heckhausen and Schulz, 1995, p. 286). Think for instance about a worker getting older and who changes his/her preferences from extrinsic (competition with younger colleagues, promotions, etc.) to more intrinsic motives (attractive job features, such as enjoyment of social contacts and learning opportunities; see also Rhodes, 1983; Kanfer and Ackerman, 2004; Kooij et al., 2011; Truxillo et al., 2012a, b). Following this line of reasoning, we argue that especially for the over-forties intra-organizational networking, being an important HRM practice, is positively related with an increase in their employability, and as a result, with their individual task performance.

To conclude, although older workers usually have less human resource developmental opportunities (Maurer et al., 2003; Martini and Cavenago, 2017), building upon life-span developmental theories and previous empirical work, we hypothesize that when older employees participate in intra-organizational networks, they will benefit relatively more in terms of their career potential, which will be depicted in higher scores for self-rated employability, and, as a result, for individual task performance. Therefore, the following hypothesis has been formulated:

*Hypothesis 5*: Employee age will moderate the mediated relationship between intra-organizational networking and individual task performance, via employability, such that this relationship will be stronger for older employees (≥40 years) in comparison with their younger counterparts (<40 years).

## Methodology

### Sample and Procedure

The data was collected with the help of 10 master students in the strategic HRM educational program offered at the Radboud University, Nijmegen, Netherlands. The students were asked to contact a variety of working organizations from their network, for instance through their parents, other relatives and friends, in order to get access to ideally 50 respondents each, across a large number of organizations. Our final sample consisted of 374 employees from a rather heterogeneous sample, working in 33 different private and public service organizations, across different occupational sectors. Anonymous on-line (using the data gathering tool Qualtrics) and paper-and-pencil versions of a survey were used. 61.76% of the respondents were female. The respondents’ average age was 37.78 (SD = 13.32). About 55.88% of the respondents were younger than 40 years old. 66.58% of the respondents had a permanent contract, 60.16% of the respondents worked part-time. The Netherlands is a country with a relatively high labor market participation rate, and, at the same time, a large number of part-time workers (Wielers and Raven, 2013). Their average tenure in the organization was 9.51 years (SD = 9.77) and 19.79% reported to occupy a supervisory position.

Table 1 shows the descriptive statistics for all variables included in the present study. In total, 0.97% of the data cells contained missing values. For the multi-item scales, means and standard deviations were computed using mean imputation. The correlations reported in Table 1 are based on “pair-wise complete observations.” In a similar vein, in the Structural Equation Models (SEM) used to test our research hypotheses, we used the “pair-wise complete observations” option. For the multi-item scales, reliability was estimated by both coefficient alpha and coefficient omega (Green and Yang, 2015) with the psych package version 1.8.4 (Revelle, 2018) in R 3.5.1 (R Core Team, 2018). Based on the values of both the alpha and omega coefficients (see Table 1, Columns 7–8), we judge the included scales as (very) reliable measures.

**TABLE 1:** Descriptives (*n* = 374).

Variable	Mean	SD	Min	Max	NA	α	ω	1	2	3	4	5	6	7	8	9	10
Network (1)	3.51	1.05	1.00	5.00	14	0.87	0.88										
Occ (2)	4.66	0.57	2.80	6.00	1	0.86	0.87	1									
Antop (3)	3.78	0.88	1.00	6.00	1	0.78	0.78	0.35	0.35								
Pflex (4)	4.61	0.58	2.40	6.00	1	0.80	0.81	0.53	0.53	0.47							
Corp (5)	4.17	0.89	1.50	6.00	1	0.82	0.82	0.37	0.37	0.61	0.56						
Bal (6)	4.19	0.70	1.75	6.00	1	0.75	0.75	0.20	0.20	0.20	0.33	0.28					
Empl (7)	4.31	0.51	2.86	5.73	1	0.90	0.93	0.66	0.66	0.76	0.80	0.82	0.54				
Perf (8)	4.07	0.55	1.56	5.00	14	0.90	0.91	0.58	0.58	0.31	0.32	0.28	0.05	0.43			
Age (9)	–	–	–	–	1	–	–	0.15	0.15	0.14	0.04	0.22	0.03	0.17	0.19		
Gender (10)	–	–	–	–	0	–	–	−0.10	−0.10	−0.14	0.01	−0.08	−0.06	−0.11	−0.03	−0.13	
Edu (11)	–	–	–	–	0	–	–	0.18	0.18	0.30	0.17	0.24	−0.03	0.25	0.20	0.11	−0.02

*Mean, Mean observed answering score; SD, standard deviation of the observed answering score; Min, Lowest observed answering score; Max, Highest observed answering score; NA, Number of respondents who skipped >40% of the items of the scale; α, coefficient alpha; ω, coefficient omega; Network, Intra-organizational networking; Occ, Occupational expertise; Antop, Anticipation and optimization; Pflex, Personal flexibility; Corp, Corporate sense; Bal, Balance; Empl, Employability (i.e., the observed average sum score on all of the five employability scales); Perf, Individual Task performance; Age, A dichotomous variable (i.e., 1: <40 years old and 2: ≥40 years old); Gender, a dichotomous variable (i.e., 1: male; 2: female); Edu (educational qualification), a dichotomous variable (i.e., 1: lower educated and 2: higher educated).*

### Measures

Intra-organizational networking was measured using the three-item scale by Bozionelos (2003). These three items assessed instrumental network resources. A sample item was: “*I personally know a number of people who occupy important posts in the organization*.” All scale anchors ranged from (1) completely disagree to (5) completely agree. Content and construct validity are good, and [see Bozionelos (2008) for predictive validity in the light of career success]. Cronbach’s alpha for this measure in this study was 0.87.

Employability was measured with the thoroughly validated short-form (Van der Heijden et al., 2018) of Van der Heijde and Van der Heijden’s (2006) instrument which consists of five scales and has proven to have sound psychometric qualities (see also Van der Heijden et al., 2009): occupational expertise (five items); anticipation and optimization (four items); personal flexibility (five items); corporate sense (four items); and balance (four items). Examples of items were: “*I consider myself competent to engage in in-depth, specialist discussions in my job domain*” (occupational expertise) [ranging from (1) “not at all” to (6) “extremely”] (Cronbach’s alpha was 0.86); “*I take responsibility for maintaining my labor market value*” (anticipation and optimization) [ranging from (1) “not at all” to (6) “to a considerable degree”] (Cronbach’s alpha was 0.78); *“I adapt to developments within my organization”* (personal flexibility) [ranging from (1) “very badly” to (6) “very well”] (Cronbach’s alpha was 0.80); “*I am involved in achieving my organization’s/department’s mission*” (corporate sense) (Cronbach’s alpha was 0.82); and “*I suffered from work-related stress”* (balance) (Cronbach’s alpha was 0.75) [both ranging from (1) “not at all” to (6) “to a considerable degree”]. Elaborate tests of its psychometric aspects, testing convergent, discriminant, and predictive validity (for career success) have yielded very promising results (see also Van der Heijden et al., 2009, 2018; Van der Heijden and Bakker, 2011; Veld et al., 2016).

Individual task performance was measured using nine items from Goodman and Svyantek’s (1999) instrument that has proven to have good psychometric qualities (see also Demerouti et al., 2014). The original scale used a seven-point answer-scale, but to increase consistency in the questionaire, we decided to measure employees’ current performance on a five-point Likert scale ranging from (1) strongly disagree to (5) strongly agree too. A sample item was: “*I achieve the objectives of the job*.” Cronbach’s alpha in this study was 0.90.

Age was measured in year of birth and for the purpose of the current study dichotomized as 1: <40 years old (“younger workers”) and 2: ≥40 years old (“over-forties”) – in line with the ADEA (Finkelstein and Farrell, 2007, p. 100; see also Boerlijst et al., 1998; Taylor and Walker, 1998 for justification for this dichotomy in research that has been conducted in Europe) – in the year in which the survey was administered.

Control variables. Given the outcomes of earlier studies, we included gender and educational qualification as control variables (Ng et al., 2005; Van der Heijden et al., 2009). Educational qualification was measured on a nine-point scale, but dichotomized for this study (i.e., 1 = lower educated, for respondents with a lower or middle-level vocational degree or with less education, 22.19% of the respondents; 2 = higher educated, for respondents with at least a degree from an applied science institute, that is a higher vocational degree, 77.81% of the respondents).

### Analyses

To test our hypotheses, we conducted a series of SEM analyses using the Lavaan package 0.6–1 (Rosseel, 2012) in R 3.5.1 (R Core Team, 2018). The annotated syntax files can be asked for from the authors of this article. As all multi-item measures contained (ordered) categorical indicators, we used the mean- and variance-adjusted weighted least squares (WLSMV) estimator to estimate the model parameters. We only report standardized regression weights. Also, we only report the robust fit measures, as differences between the robust and non-robust fit measures are negligible.

## Results

### Testing the Measurement Model

To start with, we tested the measurement model using all multi-item measures. In line with our theoretical arguments, we imposed a second-order structure on the scales tapping the five employability dimensions. A first version of our measurement model showed an adequate global fit with the data (CFI = 0.948; TLI = 0.944; RMSEA = 0.058; 90% CI RMSEA [0.054;0.063]), but produced a small negative variance estimate for one of the items tapping “balance” in the respective employability subscale [i.e., #bal4 “I achieve a balance in alternating between reaching my own work goals and supporting my colleagues” (Van der Heijden et al., 2018), variance estimate = −0.063, *p* < 0.000] and several standardized factor loadings being larger than 1.00. In order to identify the measurement model properly, we decided to constrain the unstandardized factor loading of this particular item to be smaller than 1.00, instead of dropping the item which would decrease the construct validity of the short-form employability measure (Van der Heijden et al., 2018). Adding this constraint to the model solved the estimation issues and, moreover, had no impact on the global model fit (CFI = 0.945; TLI = 0.940; RMSEA = 0.060; 90% CI RMSEA [0.056;0.065]). Hence, we decided to move forward with this slightly adjusted measurement model.

### Testing Hypotheses 1–4

Next, we tested Hypotheses 1 to 4 by extending the measurement model with the postulated regression paths. Adding the regression paths to the measurement model did not have any effect on the global model fit (CFI = 0.940; TLI = 0.935; RMSEA = 0.030; 90% CI RMSEA [0.024;0.036]). Based on our analyses, intra-organizational networking seems to be positively related to employability (β = 0.26; *p* = 0.003). Hence, Hypothesis 1 is confirmed. In contrast, Hypothesis 2 needs to be rejected using our data. In particular, the outcomes of our analyses do not suggest that intra-organizational networking is positively related to individual task performance (β = −0.09; *p* = 0.196) and neither is gender (β = −0.02; *p* = 0.728). Interestingly though, higher educated employees appear to report, on average, higher values for individual task performance (β = 0.20; *p* < 0.000). Considering Hypothesis 3, we found that employability is indeed related to individual task performance (β = 0.58; *p* < 0.000), while controlling for gender and educational background, herewith confirming our expectation. Finally, Hypothesis 4 is also confirmed with our data because of a significant indirect effect of intra-organizational network on task performance via employability (β = 0.15; *p* = 0.002, with the significance level estimated using the delta method). Given the insignificant direct effect as regards the relationship between intra-organizational networking and task performance (cf. results for Hypothesis 2), we conclude that employability fully mediates this relationship.

### Testing for Measurement Invariance

Before proceeding to Hypothesis 5, we deemed it necessary to investigate Measurement Invariance (MI) of the measurement model across both age groups (cf. Dordoni et al., 2017). After checking configural MI, we sequentially imposed more restrictions on the model parameters. In particular, we checked for weak (i.e., equal loadings), strong (i.e., equal loadings and intercepts), and strict (i.e., equal loadings, intercepts, and residuals) MI.

We note that, in a Multi-Group analysis, the WLSMV estimator cannot estimate factor loadings properly in case of sparse answering categories in the tails of the answering score distribution. To deal with this issue, we collapsed adjacent answer categories of 16 rating scale items which contained only a few or no observations in the tails of one of the age groups, so that the remaining answering categories contained sufficient respondents in both age groups.

Given the results presented in Table 2, we can expect that the strict measurement model holds for both age groups as the differences in CFI, TLI, and RMSEA of the various models are negligible. With this outcome, we can safely proceed to test our final hypothesis.

**TABLE 2:** Results for the measurement invariance tests.

					RMSEA			
	CFI	Δ CFI	TLI	Δ TLI	90% CL-LB	Mean	90% CI-UB	Δ Mean
Configural	0.932		0.927		0.055	0.060	0.065	
Weak	0.935	0.003	0.931	0.004	0.053	0.058	0.063	−0.002
Strong	0.931	−0.004	0.933	0.002	0.053	0.057	0.062	−0.001
Strict	0.931	0.000	0.933	0.000	0.053	0.057	0.062	0.000

### Testing Hypothesis 5

To test Hypothesis 5, we fitted a SEM model in which the regression weights for both age groups were estimated freely, but we added a parameter to test if the indirect effect of intra-organizational networking on individual task performance, via employability, differed for the younger versus the older workers. Given the evidence for the strict MI, we constrained the loadings, intercepts, and residuals to be equal in both measurement models. It has to be noted that we did not compare a multiple-group model in which all parameters were fixed with a multiple-group model in which all parameters were freed because such a procedure would not show us which specific structural parameters are different or equal in both groups. In particular, we considered such an analysis too strict as we did not have any hypotheses on the moderating effect of age on the other relationships in the model and, moreover, we cannot rule out that such a strict analysis – at the global level – cancels out the postulated moderated effect stated in Hypothesis 5.

The estimated multi-group mediation model has a satisfactory fit with the data (CFI = 0.958; TLI = 0.959; RMSEA = 0.020; 90% CI RMSEA [0.008;0.029]). Table 3 shows the regression results for this model. The last row of this table reveals that the hypothesized indirect effect differed to some degree for both age groups (β = 0.11, *p* = 0.065 for younger employees; β = 0.19, *p* = 0.018 for older employees). That is, the indirect effect is significant in the group of older employees, but insignificant in the group of younger employees suggesting some moderation effect. However, we – conservatively – rejected Hypothesis 5 because of the insignificant difference between both regression coefficients (*p* = 0.435, significance level estimated using the delta method)^1^. Thus, we tentatively conclude that the indirect effect of employability on the relationship between intra-organizational networking and individual task performance does not differ between younger and older employees.

**TABLE 3:** Outcomes of multi-group moderated mediation model.

	Age group < 40				Age group ≥ 40			
	β	se	*z*-value	*p*-value	β	se	*z*-value	*p*-value
**Direct effects**								
Intra-organizational networking ON employability	0.19	0.10	1.98	0.047	0.31	0.12	2.49	0.013
Intra-organizational networking ON performance	−0.14	0.08	−1.82	0.069	−0.08	0.14	−0.56	0.576
Employability ON performance	0.57	0.06	8.89	0.000	0.61	0.08	7.31	0.000
Gender ON performance	0.05	0.07	0.75	0.454	−0.03	0.08	−0.38	0.705
Education ON performance	0.27	0.07	4.08	0.000	0.08	0.08	0.95	0.341
**Indirect effects**								
Intra-organizational networking ON Performance VIA Employability	0.11	0.06	1.84	0.065	0.19	0.08	2.37	0.018

In addition, we note that neither the other effects differed substantially between both age groups, with the exception being educational qualification. The moderation analysis reveals that the effect of educational background on individual task performance is significant for younger employees, but not for older employees (β = 0.27 for younger employees; β = 0.08 for older employees), suggesting that effect of educational background tapers off when employees grow older (cf. the results for Hypothesis 2).

## Discussion

### Outcomes and Implications of the Test of the Multi-Group Mediation Model of Individual Task Performance Enhancement

In this study, we adopted a human capital perspective (Becker, 1993) and life-span development theories [SOC theory; Baltes et al., 1999 and Life-Span Theory of Control (Heckhausen et al., 2010)] to investigate the effect of intra-organizational networking on individual task performance, via employability; and if so, whether or not this effect differs for younger (<40 years old) versus older employees (≥40 years old) using a sample of 374 employees working in a range of different private and public service organizations, across different occupational sectors. Based on a series of various SEM analyses, we conclude that, in line with our expectations, employability fully mediates the relationship between intra-organizational networking, but that, unexpectedly, this effect did not vary between younger and older employees. The finding that employability fully mediated the relationship between intra-organizational networking and individual task performance, underscores that social capital has its impact on the employee’s performance at work through their increased competences or career potential. This implies that working organizations need to be continuously concerned with the sustainability of their workers’ employability in order to safeguard their performance across their career (Savickas, 2005; Martini and Cavenago, 2017; De Vos et al., 2020; Van der Heijden et al., 2020).

The outcomes of our multi-group analysis indicate that our age moderation hypothesis could not be confirmed in this study. We note that while interpreting the regression results we found the indirect effect to be significant in the group of older employees, but insignificant in the group of younger employees suggesting some moderation effect. However, after comparing the unconstrained multiple-group model with a model wherein we constrained the regression coefficients for intra-organizational networking on employability as well as the regression coefficients for intra-organizational networking and employability on performance, we needed to conclude that there are no significant differences between both groups. Hence, the moderation hypothesis should be rejected. These outcomes are interesting and might lead us to think that, regardless of age, intra-organizational networking is a key mechanism for knowledge transfer that fosters close cooperation of colleagues across organizational units, herewith facilitating the capacity to absorb knowledge (Van Wijk et al., 2008). Obviously, a sound knowledge base that is kept up to date is highly necessary to protect one’s employability across the life-span (Quendler and Lamb, 2016). In line with Li et al. (2020), who adopted an intellectual capital perspective, we argue that age-diverse workforces might be of high value for nowadays’ working organizations, in case both workers and their employers protect and foster the continuous development of occupational competencies throughout their working life.

Nevertheless, more research is needed using larger samples to investigate whether our particular outcome might be attributed to the relatively small sample size of both groups. Furthermore, a conceivable explanation for this unexpected finding may lie in a possible non-linear moderation effect. More specifically, the hypothesized moderation effect of age might be non-linear, occurring only at a certain cut-off point, such as 50 or 55 years of age [cf. Armstrong-Stassen and Schlosser (2008) in their research on the role of job development climate in the retention of older workers above the age of 50; see also Van der Heijden et al., 2016]. It is also thinkable that different conceptualizations of employee age might result into significant age moderation effects (see for instance Sterns and Doverspike, 1989) categorization into (chronological age, functional or performance-based age, psychosocial or subjective age, organizational age, and the concept of life-span age). Kooij et al. (2011) already found that different conceptualizations of age have different effects on work-related outcomes.

### Limitations of the Study

Besides the strengths of our current study (in terms of being the first scholarly work that considers the importance of networking as a possible antecedent in the light of employability across the life-span, and through this, on performance at work), the present study has some limitations. Firstly, all data have been collected using survey research, herewith opening up the possibility of response set consistencies. Secondly, although we have used control variables in order to optimize the use of our design (see Spector, 2019 for more details), the study is cross-sectional, which implies that further research is needed in order to address issues of causality (see also Bono and McNamara, 2011 on the constraints of using cross-sectional data for testing a mediation model). Thirdly, like most studies in our field, all findings are based upon reports from a single source only, namely, employees’ perceptions. In this respect, we followed Podsakoff et al. (2003) and guaranteed anonymity, herewith encouraging respondents’ openness. In addition, we used SEM analyses and have investigated all model relationships simultaneously. The outcomes of Harman’s single factor test indicated that the incorporated measures were clearly separated from each other. Therefore, we are confident that possible common-method effects do not downplay the significance of our results to any serious extent. A final concern could be that particularly self-reports for performance entail some confounding effects, for instance the tendency for employees to over-rate their performance (i.e., the lenience effect; Tsui and Ohlott, 1988). However, as our sample mainly included higher educated workers, the threat associated with biased performance judgments is assumed to be limited (see also Kruger and Dunning, 1999; David et al., 2006; De Cuyper and De Witte, 2011).

### Recommendations for Future Research

Research using multi-wave designs can increase our knowledge about the stability and change of the model variables, and about cross-lagged (i.e., over time) relationships (Taris and Kompier, 2003; De Lange et al., 2004; Spurk and Abele, 2014). For example, it might be that more employable workers are also more actively searching for, and participating in, social networks, or get more opportunity to do so (think about the Matthew effect). As a result, they might even become more employable, which would imply positive reciprocal relationships between the two model variables. In addition, it might also be the case that high task performance increases an individual worker’s employability. We would also like to call for more research incorporating more refined age breakdowns, in order to better understand life-span developmental processes (see for instance Van der Heijden, 2002 who differentiated between starters, middle-aged, and seniors) (see also Sullivan and Crocitto, 2007). More research is also needed to investigate the generalizability of our findings to other countries, that is to say, whether they are culturally invariant, and to examine possible effects of occupational sector/job type and job type. In addition, future research incorporating different evaluation perspectives (for instance using 360-degree feedback methodology) (Hensel et al., 2010) may add incremental validity to the measurement of individual task performance. The competence-based measure of employability that has been used in this study is definitely suitable to investigate one’s capacity to perform well at tasks in other potential jobs inside one’s current organization or with an alternative employer as well. Finally, as employability enhancement is a multi-facetted endeavor, which should consider personal and contextual factors (see Van der Heijden and Spurk, 2019), it is interesting to conduct more research incorporating further mediators and/or moderators. Another interesting avenue for future scholarly work comprises examining the predictive validity of the separate employability dimensions in the light of individual task performance. Preferably, research in this area also incorporates alternative work performance measures, such as productivity, quality of the employee’s output, velocity, to mention but a few. Last but not least, it is of utmost importance to gain more insight into how COVID-19 has influenced the relationships between our model variables, for instance as a result of the increased amount of teleworking, herewith including educational background, age, gender as possible important factors as well.

### Practical Implications

This contribution stresses the importance of intra-organizational networking, both in terms of workers’ own employability as well as in the light of the added value for their organization, that is, in terms of their performance at work. Moreover, this study portrays that its importance is similar for men and women, and for younger and olders staff members alike. In particular, participation in intra-organizational networks enhances career prospects, which is highly beneficial for both the individual employee, in terms of increased career prospects. Such network resources increase the individuals’ confidence in their ability to survive the organization’s current and future requirements. Moreover, network ties within the organization have positive consequences for the employer as well, as these ties entail career success and organizational commitment (see also Bozionelos, 2008). Therefore this study implies that a better understanding of individual employees’ motives and how they are related to networking is crucial for stakeholders in working organizations in order to improve their staff members performance at work. Given the fact that implicit motives operate without awareness, individuals need to consciously focus on and regulate their networking behaviors (Wolff et al., 2018), and search for jobs that provide ample networking opportunities. Obviously, given the dual responsibility for employability enhancement (Van der Heijden and De Vos, 2015) their direct manager, HR representatives and training and development specialists play an important role in this regard as well. Concrete, the parties involved might consider using incentives to motivate individuals to proactively initiate behaviors aimed at building, developing and maintaining networks. Because, nowadays, job qualifications are changing and becoming increasingly more complex, building networks of relationships in working organizations that can help further the individual’s career progression (Bozionelos, 2008) is an important key to protect and further enhance one’s sustainability throughout the life-span (Van der Heijden and De Vos, 2015; De Vos et al., 2020).

## Data Availability Statement

The raw data supporting the conclusions of this article will be made available by the authors upon request, without undue reservation.

## Ethics Statement

Ethical review and approval was not required at the time the study was performed in accordance with the local legislation and institutional requirements. The patients/participants provided their informed consent to participate in this study.

## Author Contributions

BV, PK, and GN worked on the design, modeling, analyses, and writing of the manuscript. All authors contributed to the article and approved the submitted version.

## Conflict of Interest

The authors declare that the research was conducted in the absence of any commercial or financial relationships that could be construed as a potential conflict of interest.

## Footnotes

1 We also compared the unconstrained multiple-group model with a model wherein we constrained the regression coefficients for intra-organizational networking on employability as well as the regression coefficients for intra-organizational networking and employability on performance to be equal in both age groups. These constraints did not result in a significant worse model. Indeed, with a X^2^(6) = 6.73, *p* = 0.35, we must conclude that there are no significant differences between both groups. Hence, the moderation hypothesis should be rejected.
